# Identification of Mimotopes with Diagnostic Potential for *Trypanosoma brucei gambiense* Variant Surface Glycoproteins Using Human Antibody Fractions

**DOI:** 10.1371/journal.pntd.0001682

**Published:** 2012-06-12

**Authors:** Liesbeth Van Nieuwenhove, Philippe Büscher, Fatima Balharbi, Michael Humbert, Tessa Dieltjens, Yves Guisez, Veerle Lejon

**Affiliations:** 1 Department of Biomedical Sciences, Institute of Tropical Medicine, Antwerp, Belgium; 2 Dana-Farber Cancer Institute, Boston, Massachusetts, United States of America; 3 Harvard Medical School, Boston, Massachusetts, United States of America; 4 Department of Biology, University of Antwerp, Antwerp, Belgium; IRD/CIRDES, Burkina Faso

## Abstract

**Background:**

At present, screening of the population at risk for *gambiense* human African trypanosomiasis (HAT) is based on detection of antibodies against native variant surface glycoproteins (VSGs) of *Trypanosoma brucei* (*T.b.*) *gambiense*. Drawbacks of these native VSGs include culture of infective *T.b. gambiense* trypanosomes in laboratory rodents, necessary for production, and the exposure of non-specific epitopes that may cause cross-reactions. We therefore aimed at identifying peptides that mimic epitopes, hence called “mimotopes,” specific to *T.b. gambiense* VSGs and that may replace the native proteins in antibody detection tests.

**Methodology/Principal Findings:**

A Ph.D.-12 peptide phage display library was screened with polyclonal antibodies from patient sera, previously affinity purified on VSG LiTat 1.3 or LiTat 1.5. The peptide sequences were derived from the DNA sequence of the selected phages and synthesised as biotinylated peptides. Respectively, eighteen and twenty different mimotopes were identified for VSG LiTat 1.3 and LiTat 1.5, of which six and five were retained for assessment of their diagnostic performance. Based on alignment of the peptide sequences on the original protein sequence of VSG LiTat 1.3 and 1.5, three additional peptides were synthesised. We evaluated the diagnostic performance of the synthetic peptides in indirect ELISA with 102 sera from HAT patients and 102 endemic negative controls. All mimotopes had areas under the curve (AUCs) of ≥0.85, indicating their diagnostic potential. One peptide corresponding to the VSG LiTat 1.3 protein sequence also had an AUC of ≥0.85, while the peptide based on the sequence of VSG LiTat 1.5 had an AUC of only 0.79.

**Conclusions/Significance:**

We delivered the proof of principle that mimotopes for *T.b. gambiense* VSGs, with diagnostic potential, can be selected by phage display using polyclonal human antibodies.

## Author Summary

Control of the chronic form of sleeping sickness or *gambiense* human African trypanosomiasis (HAT) consists of accurate diagnosis followed by treatment. We aim to replace the native variant surface glycoprotein (VSG) parasite antigens that are presently used in most antibody detection tests with peptides that can be synthesised *in vitro*. Antibodies recognising VSG were purified from HAT patient sera and were used to select phage-expressed peptides that mimic VSG epitopes from a Ph.D.-12 phage display library. The diagnostic potential of the corresponding synthetic peptides was demonstrated in indirect ELISA with sera from HAT patients and endemic negative controls. We proved that diagnostic mimotopes for *T.b. gambiense* VSGs can be selected by phage display technology, using polyclonal human antibodies.

## Introduction

The chronic form of sleeping sickness or human African trypanosomiasis (HAT) in West and Central Africa is caused by the protozoan parasite *Trypanosoma brucei (T.b.) gambiense* while *T.b. rhodesiense* causes a more fulminant, acute form in East and Southern Africa. Both subspecies of *T. brucei* are cyclically transmitted by tsetse flies of the genus *Glossina* and mainly affect poor, rural populations. The true burden of this disease is unknown as many cases remain undiagnosed or unreported [1], [2].

Since untreated HAT is almost always fatal and no inexpensive, safe and easily administered drugs are available, accurate case detection is crucial. Parasite detection is laborious and insensitive, and remains therefore limited to disease suspects. In the absence of reliable clinical symptoms or antigen detection tests, HAT suspects are identified through screening of the population at risk for presence of trypanosome specific antibodies. The commonly used antibody detection tests, card agglutination test for trypanosomiasis (CATT) [3], LATEX/*T.b. gambiense* and ELISA/*T.b. gambiense* [4], [5] detect antibodies against the highly immunogenic variant surface glycoproteins (VSGs) of *T.b. gambiense*. Even though the genome of *T. brucei* contains >1000 VSG genes, only one variable antigen type (VAT) is expressed at a time. Stochastic switching of VSG allows the trypanosome to evade the specific antibody responses that were raised against earlier VATs [6]–[10]. Some VATs, such as LiTat 1.3 and 1.5, are recognised by almost all *gambiense* HAT patients and therefore called predominant. The dense VSG monolayer on the living trypanosome shields all non-specific epitopes. The hypervariable N-terminal VSG domain (300–400 residues) is exposed to the immune system and comprises the VAT-specific epitopes, while the relatively conserved C-terminal domain (40–80 residues) is hidden by the intact VSG coat [6], [9], [11], [12].

Disadvantages of the present antibody detection tests include the occurrence of non-specific reactions. This might be explained by exposure of non-HAT-specific epitopes that are normally shielded on the living trypanosome [12], [13]. In addition, diagnostic test production actually requires culture of infective *T.b. gambiense* in large numbers of laboratory rodents and poses an important risk of infection to the manufacturing staff [14].

These drawbacks can be circumvented through the use of synthetic peptides that mimic HAT-specific VSG epitopes (mimotopes) and can be produced in a standardised way [15]. One way to identify such mimotopes is by peptide phage display. This technique is based on DNA recombination resulting in foreign peptides with random sequences that are displayed fused to the pIII surface protein of the M13 phage. After an *in vitro* selection process based on binding affinity and several rounds of enrichment (panning), the encoded peptide insert sequence of the selected phage is deduced from the phage DNA. We previously reported successful identification of mimotopes for VSG LiTat 1.3 and LiTat 1.5 by performing phage display with three monoclonal antibodies [16]. However, by the use of only three monoclonal antibodies, representing only a fraction of the VSG-specific antibody response, some mimotopes with diagnostic potential might have been missed. Additionally, the mouse and human immune system may recognise different B cell epitopes. The use of polyclonal human antibodies might therefore increase chances of selecting diagnostic mimotopes [17]. Polyclonal antibodies from human sera have been previously used for selection of mimotopes with diagnostic potential for e.g. hepatitis C [15], typhoid fever [18] and Epstein Barr virus [17]. Some mimotopes have been patented for incorporation in commercially available tests, e.g. for neurocysticercosis [19].

In this manuscript we describe the identification of mimotopes for VSG LiTat 1.3 and LiTat 1.5 through phage display, using sera from HAT patients and endemic negative persons.

## Materials and Methods

### Ethics statement

Sera from HAT patients and endemic controls were collected within different diagnostic studies [5], [20]. All individuals gave their written informed consent before providing blood. Permission for these studies was obtained from the national ethical committee of the Democratic Republic of the Congo (DR Congo) and from the Institute of Tropical Medicine Antwerp (ITMA) ethical committee, reference number 03 07 1 413 and 04 44 1 472. Forty additional endemic negative control specimens were obtained from the archived specimen bank of the Parasite Diagnostics Unit at ITMA. All specimens were anonymised.

### Coating of magnetic particles with VSG LiTat 1.3 or LiTat 1.5

Variant surface glycoproteins were purified from cloned populations of *T.b. gambiense* Variable Antigen Type (VAT) LiTat 1.3 and 1.5 [4]. VSG LiTat 1.3 or LiTat 1.5 were coated onto magnetic particles (MP, Estapor, 10% suspension, 1.04 µm, 9 µeq/g COOH). A volume of 250 µL of MP suspension was washed twice with 1 mL of buffer A (10 mmol/L NaH_2_PO_4_, pH 6.0). The MP were activated with 2.5 ml of buffer A containing 25 mg of 1-ethyl-3-(3-dimethylaminopropyl) carbodiimide (Pierce) and 15 mg of N-hydroxysuccinimide (Sigma). The MP were rotated for 15 minutes at room temperature (rT) and washed with 1 mL of buffer B (2 mmol/L HCl) where after 350 µg of VSG (LiTat 1.3 or LiTat 1.5) in 1.5 mL of buffer C (20 mmol/L NaH_2_PO_4_/Na_2_HPO_4_, pH 7.5) was added to the pellet of MP. After rotating for 2 h at rT, the MP were washed three times with buffer C and resuspended to a final concentration of 8% in buffer C containing 100 mmol/L glycin, 1% bovine serum albumin (BSA) and 0.1% NaN_3_. Successful coating of the MP was evaluated by agglutination with a HAT positive serum diluted 1/4 in phosphate buffered saline (PBS, 0.01 mol/L phosphate, 0.14 mol/L NaCl, pH 7.4) containing 0.02% w/v NaN_3_.

### Affinity purification of VSG LiTat 1.3 or LiTat 1.5 specific antibodies from HAT serum

Antibodies specific to VSG LiTat 1.3 or LiTat 1.5 were purified from nine HAT positive sera originating from the DR Congo [20]. One mL of LiTat 1.3 or LiTat 1.5 coated MP was rotated for 2 h at rT with 125 µL of HAT positive serum. After five washes with 800 µL of PBS, the specific antibodies were eluted from the MP by adding 700 µL of 0.2 mol/L glycine/HCl (pH 2.2) followed by magnetic separation after five minutes. The eluates, corresponding to the affinity purified antibody fractions, were neutralised with 100 µL of 1 mol/L Tris/HCl pH 9.1.

### Indirect ELISA on VSG

Indirect ELISA was used to screen the affinity purified antibody fractions and all human serum samples on reactivity with VSG LiTat 1.3 and LiTat 1.5. ELISA plates (Nunc MaxiSorp™) were coated overnight (ON) at 4°C with 100 µL/well of 2 µg/mL of each VSG separately in phosphate buffer (PB, 0.01 mol/L phosphate, pH 6.5) or with 1.7 10^11^ particle/mL of wild type phage (WTP) in PBS. One plate was left empty as antigen negative control (Ag0). The plates were tapped dry, saturated with 350 µL/well of PBS-Blotto (0.01 mol/L phosphate, 0.2 mol/L NaCl, 1% w/v skimmed milk powder, 0.05% w/v NaN_3_) during 1 h at rT and washed three times with 0.05% v/v Tween-20 in PBS (PBST) (ELx50, Bio-Tek ELISA washer). The purified antibody fractions were diluted 1/25 and human serum samples 1/150 in PBS-Blotto. One hundred µL/well of each dilution was applied in duplicate and incubated for 1 h at rT. After three washes with PBST 100 µL/well of horse radish peroxidase (PO)-conjugated goat anti-human IgG (H+L) (Jackson), 1/40000 diluted in PBST, was added. An hour and five washes later, wells were incubated for 1 h at rT with 100 µL/well of 2.2′-azino-bis-(3-ethylbenzthiazoline-6-sulfonic acid) (ABTS) chromogen/substrate solution (50 mg tablet/100 mL of ABTS buffer, Roche). The plate was shaken for ten seconds and the optical density (OD) was read at 414 nm (Labsystems Multiskan RC 351). The measured OD was corrected (OD_c_) with the corresponding OD in the Ag0 wells.

### Coating of magnetic particles with human IgG antibodies

Three LiTat 1.3 positive pools, each consisting of three different VSG LiTat 1.3-specific antibody fractions, three LiTat 1.5 positive pools, each consisting of three different VSG LiTat 1.5-specific antibody fractions and one negative pool of four endemic negative sera were prepared. For each pool the antibodies were coated onto anti-human IgG (H+L) functionalised magnetic particles (MP) (1% w/v, 1.05 µm, Estapor/Merck) according to the guidelines of the manufacturer.

### Selection of mimotopes for VSG LiTat 1.3 and LiTat 1.5 by panning of phage-displayed peptides

The panning was performed with the Ph.D.-12 (12-mer) phage display library (New England Biolabs, NEB) [21] through two rounds consisting of 1) a positive selection with anti-VSG (LiTat 1.3 or 1.5, respectively) antibodies coated on MP, 2) a negative selection with endemic negative serum antibodies coated on MP and 3) phage amplification [22]. Each positive selection was followed by phage titration and sandwich ELISA. After these two rounds a third positive selection was performed.

### Positive selection

Positive selection was performed as previously described [16]. Bound phages were eluted for ten minutes with 600 µL of 0.2 mol/L glycine-HCl containing 1 mg/mL BSA (pH 2.2) and neutralised with 90 µL of Tris-HCl (1 mol/L, pH 9.1).

### Negative selection

Six hundred µL of the elution from the positive selection was rotated ON at 4°C with 1 mg of MP coated with endemic negative serum antibodies, in a total volume of 1 mL of PBSG.

### Amplification and purification of phages

The unbound phages in 900 µL of the supernatant of the negative selection were amplified, in a culture of *Escherichia (E.) coli* (strain ER2738, NEB) at early log (0.01–0.05 A_600_), and purified with PEG-NaCl as previously described [16], [21].

### Titering of phages

Phages from the first, second and third positive selection were diluted in PBS 10^1^ to 10^4^, 10^2^ to 10^5^, 10^4^ to 10^7^, respectively. Ten µL of these dilutions were incubated for five minutes with 200 µL of an *E. coli* culture in mid-log (0.4–0.5 A_600_). The mixture was then pipetted into 4 mL of Top-Agar (50°C) and poured onto agar plates containing 1 mL/L IPTG/X-gal (1.25 g isopropyl β-D-thiogalactoside, 1 g 5-bromo-4-chloro-3-indolyl-β-D-galactoside, 25 mL dimethylformamide); ninety-four blue clones were picked and each clone was inoculated in 200 µL of lysogeny broth (LB) in a sterile culture plate (BD Falcon™ Clear 96-well Microtest™ Plate) [21]. This plate was shaken overnight at 30°C, and then the bacteria were pelleted by 5 min centrifugation at 1312 g. The supernatant was tested in a sandwich ELISA.

### Sandwich ELISA with phage particles

ELISA plates were coated ON at 4°C with 100 µL/well of VSG LiTat 1.3- or LiTat 1.5-specific positive antibody pools (5 µg/mL in PBS) or a 1/10000 dilution in PBS of the negative serum pool. The ELISA was performed as previously described [16]. Briefly, the wells were incubated for 1 h at rT with 100 µL of phage dilution in PBS-Blotto (1/3 for culture plate supernatant or 1/20 for PEG-NaCl purified phage). PO-anti-M13 pVIII mAb (GE Healthcare), diluted 1/2000 in PBST was added to the wells for 1 h at rT. The wells were then incubated for 1 h at rT with ABTS and read at 414 nm.

Phage clones were withheld after the first two positive selections if 1) the OD with the corresponding positive pool (OD_pos_)>average OD_pos_+2*standard deviation (sd_pos_) and 2) the OD with the negative pool (OD_neg_)<average OD_neg_.

After the third positive selection, phages were sequenced if 1) OD_pos_>average OD_pos_+1* sd_pos_ with at least one of the positive pools, 2) OD_pos_ with the 3^rd^ positive pool >0.150 or 0.200 for phages selected for VSG LiTat 1.3 or LiTat 1.5 respectively and 3) OD_neg_<average OD_neg_. Withheld phage clones were sequenced and tested in a similar sandwich ELISA with as capture antibody the nine individual affinity purified antibody fractions, diluted 1/70 in PBS.

### Single-stranded DNA extraction, sequencing and sequence analysis

Purification of phage DNA was performed according to the NEB manual [21]. Sequence determination was performed as described before [16]. The obtained sequence chromatograms were read with Chromas 2.33 (Technelysium Pty Ltd). Sequence alignment was performed manually and with RELIC software [23]. A protein data base (pdb) model of the N-terminal domain of VSG LiTat 1.5, was created using SWISS-MODEL [24], [25]. Modelling was based on the known structure of VSG MITat 1.2 (pdb 1vsgA), previously derived by X-ray crystallography [26]. For VSG LiTat 1.3 however the server could not find a template with sufficient sequence homology, hence the pdb was created by Thomas Juetteman from the PyMol helpdesk (PyMOL Molecular Graphics System, Schrödinger, LLC). In order to identify possible conformational epitopes, the 3D-Epitope-Explorer (3DEX) [27] was used to find structural homology between the mimotope sequences and the respective VSG protein sequence.

Molecular graphics images were produced using the UCSF Chimera package from the Resource for Biocomputing, Visualization, and Informatics at the University of California, San Francisco (supported by NIH P41 RR001081) (http://www.cgl.ucsf.edu/chimera).

### Peptide synthesis

The peptides were synthesised at >85% purity (Peptide 2.0, Chantilly, VA, U.S.). The GGGS-spacer, separating the library insert and the pIII phage protein, was added to the C-terminus of the peptides that were selected by phage display [16], [21]. All peptides were C-terminally elongated with an additional lysine-biotin and amidated (-CONH_2_), mimicking the uncharged peptide bond in a protein. All synthetic peptides were reconstituted in sterile deionised H_2_O to a concentration of 2 mg/mL.

### Indirect ELISA on biotinylated synthetic peptides and human sera

First, the reactivity of all biotinylated synthetic peptides was evaluated with the nine sera used for affinity antibody purification and nine endemic negative controls. Second, the diagnostic performance of the synthetic peptides was evaluated with human serum samples that were previously screened (indirect ELISA on VSG, serum dilution 1/100) on reactivity with VSG LiTat 1.3 and 1.5. All 102 serum samples from *gambiense* HAT patients originated from DR Congo [20]. Of the 102 endemic *gambiense* HAT negative serum samples, 71 originated from the DR Congo and 31 from Benin. The indirect ELISA on biotinylated peptides was performed as the indirect ELISA on VSG but 150 µL/well was applied in all but the saturation and washing steps. ELISA plates were coated with 10 µg/mL streptavidin (NEB) in carbonate buffer (0.1 mol/L, pH 9.2) or with 2 µg/mL VSG LiTat 1.3 and LiTat 1.5 in PB, or wells were left empty (Ag0). After saturation with PBS-Blotto, the peptides were added at a concentration of 2 µg/mL in PBS to the wells containing streptavidin. The peptide-free wells received only PBS. To the VSG-containing and Ag0 wells PBS-5% w/v sucrose was added. After incubation of 1 h at rT the plates were tapped dry, sealed and frozen at −80°C. The serum samples were centrifuged for 5 min at 15700 g and diluted 1/100 in PBS-Blotto. After thawing of the plates and three washes with PBST, the serum dilutions were applied in duplicate. After one hour, we added PO-conjugated goat anti-human IgG (H+L), 1/40000 diluted in PBST. ABTS was used as chromogen/substrate solution and the OD was read as described above. The measured OD was corrected by subtracting the corresponding OD in the peptide-free or Ag0 wells and the average of the duplicate corrected ODs was taken (OD_c_).

The accuracy of the synthetic peptides to detect VSG-specific antibodies for diagnosis of sleeping sickness was assessed by the area under the receiver operator characteristics (ROC) curve (AUC) [28]. Confidence intervals were calculated according to DeLong [29]. For the whole range of cut-offs the Youden index was determined (Youden index = sensitivity+specificity−1) [30] and the cut-off with maximal Youden index was retained.

## Results

### Affinity purification of VSG-specific antibodies

In indirect ELISA, all affinity purified antibody fractions reacted specifically with their corresponding VSG and not with WTP. The antibody fractions that were purified with VSG LiTat 1.3, had an average OD_c_ of 0.533±0.319 with VSG LiTat 1.3, and average OD_c_s of only 0.032±0.032 with VSG LiTat 1.5 and −0.008±0.015 with WTP. The antibody fractions purified with VSG LiTat 1.5 had an average OD_c_ of 1.406±0.487 with VSG LiTat 1.5, and average OD_c_s of only 0.037±0.064 with VSG LiTat 1.3 and −0.017±0.018 with WTP.

The negative serum samples did not react with VSG LiTat 1.3 (OD_c_ 0.034±0.078), nor with VSG LiTat 1.5 (OD_c_ 0.028±0.069), nor with WTP (OD_c_ 0.013±0.029).

### Selection of mimotopes for VSG LiTat 1.3

During the selection process, none of 94 phage clones of the first positive selection, eight of 188 phage clones of the second positive selection and 11 of 188 phage clones of the third positive selection reacted in the sandwich ELISA and were sequenced, resulting in 18 sequences (table 1).

**Table 1:** Peptide sequences of phage clones selected with human anti-VSG LiTat 1.3 antibodies.

Pos selection	Phage clone	Peptide sequence	OD_c_ (average ± SD)	Synthetic peptide
2	3-2-E2	WDSDCKRSCRVH	0.755±0.924	3-2-E2
2	3-2-G5	LTWVSDSKSGNT	0.530±0.528	3-2-G5
2	3-2-G10	TIAPSWATDSKP	0.449±0.417	3-2-G10
2	3-2-C5	TPNNAQKQPQLP	0.444±0.533	3-2-C5
2	3-2-B12	SWMPDSKVFASH	0.383±0.394	*
2	3-2-D10	WETDQKFKQRVA	0.342±0.347	3-2-D10
2	3-2-D11	WETDQKFKQRVA	na	/
2	3-2-F6	SAYDDVKRFYTN	0.307±0.683	/
3	3-3-F6	ETDNMKPLHLRQ	0.648±1.001	3-3-F6
3	3-3-E3	VNDASKLFYPRS	0.386±0.510	3-3-E3
3	3-3-H3(1)	WPTSWHMWLANR	0.161±0.146	/
3	3-3-A7	GVPDNHKPARTQ	0.131±0.121	/
3	3-3-A2	ALPTHMNWVMPV	0.113±0.074	/
3	3-3-A4	TWPQWWWTNSKG	0.111±0.071	/
3	3-3-B8	NPPIWGTATKGI	0.091±0.058	/
3	3-3-E8	FWKPHRTHFWWG	0.080±0.046	/
3	3-3-H3(2)	YNWETDKPMPVP	0.051±0.034	/
3	3-3-A3	TWWWHSLAKTPH	0.036±0.020	/
3	3-3-C12	TTWNFKHWWPYR	0.021±0.018	/

3-3-H3(1) and 3-3-H3(2) are two different phage clones, na: not applicable, SD: standard deviation,

*: not withheld: similar to 3-2-G5 & 3-2-G10.

The phage clones were selected after two or three positive (pos) selections. The peptide sequences that were expressed by these phage clones are given in column three. The average OD_c_ in sandwich ELISA, using nine purified antibody fractions as capture antibody, is shown in column four. If the peptide sequence was synthesised, the name of the biotinylated peptide is given in column five.

**Figure 1:**
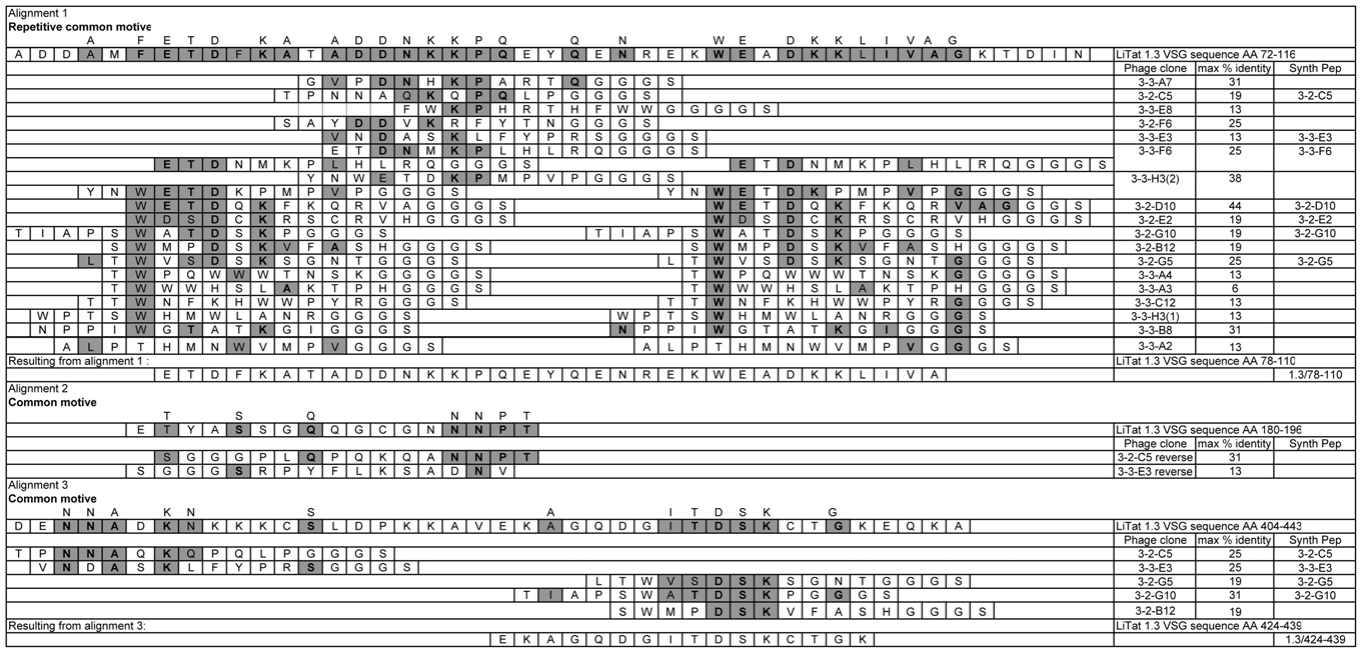
Alignment on VSG LiTat 1.3 of peptides selected with anti-VSG LiTat 1.3 antibody fractions. Homologous sequences between phage displayed peptides and/or the protein sequence of VSG LiTat 1.3 are indicated in grey. Amino acids that are identical to those of the VSG protein sequence are in bold and grey. All peptide sequences include the GGGS-spacer at the C-terminus. Maximum % identity: percentage identity of the peptide sequence with a corresponding stretch of sixteen AA within the protein sequence of VSG LiTat 1.3. Synth peptide: name of the synthesised peptide.

The alignment results of VSG LiTat 1.3 [GenBank AJ304413] and the eighteen peptide sequences displayed by the phage clones are presented in figure 1. All peptides could be aligned within amino acid stretch (AA) 72 to 116 of the N-terminal domain of VSG LiTat 1.3 (alignment 1). The common motive (F/W)ExDxK(A/V/L)x(A/V/L) was repeated twice in this VSG AA stretch. Therefore twelve sequences could be aligned twice within this region. The peptide displayed by phage 3-3-F6, ETDNMKPLHLRQ, could even be aligned three times within this region of VSG LiTat 1.3, having ETD, DNxKP and ExD identical within amino acids 78 to 80, 87 to 91 and 102 to 104 of the protein sequence. The peptide sequence displayed by phage 3-2-D10 had the highest identity within AA 72 to 116 of the VSG LiTat 1.3 sequence (7/16 AA, 44%). The reverse sequence of the peptides displayed by phage clones 3-2-C5 and 3-3-E3 showed respectively 31 and 13% identity within AA 180 to 196 (alignment 2). Within the C-terminal domain (alignment 3), the peptide expressed by phage clone 3-2-C5 and 3-3-E3 had 25% identity (4/16 AA) within AA 404 to 443 of VSG LiTat 1.3. The peptide expressed by phage clones 3-2-G10, 3-2-G5 and 3-2-B12 were respectively 31%, 19% and 19% identical within AA 404 to 443 of VSG LiTat 1.3.

All selected phage clones were tested in a sandwich ELISA with the individual purified antibody fractions. The peptides displayed by the seven phage clones with the highest average OD_c_ were withheld for synthesis as biotinylated peptides (table 1). The peptide displayed by phage clone 3-2-B12 was not withheld, since it was a homologue of 3-2-G10 and 3-2-G5 but had a lower average OD_c_. Based on the alignment results, AA stretch 78 to 110 and AA stretch 424 to 439 of the protein sequence of VSG LiTat 1.3 were also synthesised as biotinylated peptides (respectively peptide 1.3/78-110 and peptide 1.3/424-439).

The reactivity of all nine biotinylated synthetic peptides was evaluated in indirect ELISA with the nine HAT positive sera used for affinity antibody purification, and with nine endemic negative controls. Peptide 1.3/78-110 was the best performing peptide with OD_c_ 1.469. Peptide 1.3/424-439 gave a lower average OD_c_ (0.246) than peptides 3-2-G10 and 3-2-G5 (0.564 and 0.920), sharing the same common motive, and was not withheld for further testing. Peptide 3-2-E2, a homologue of peptide 3-2-D10, also gave a lower average OD_c_ (0.541 versus 0.763) and was also not withheld for testing on diagnostic performance.

### Selection of mimotopes for VSG LiTat 1.5

During the selection process, one of 94 phage clones of the first positive selection, two of 94 phage clones of the second positive selection and 20 of 188 phage clones of the third positive selection reacted in the sandwich ELISA and were sequenced, resulting in 20 sequences (table 2).

**Table 2:** Peptide sequences of phage clones selected with human anti VSG LiTat 1.5 antibodies.

Positive selection	Phage clone	Peptide sequence	OD_c_ (average ± SD)	Synthetic peptide
1	5-1-F9	AAIMHQEQESNT	0.403±0.693	5-1-F9
2	5-2-D3	SAGFENDGTKLA	0.246±0.120	5-2-D3
2	5-2-H2	TGLPTTNKQTSS	0.234±0.196	5-2-H2
3	5-3-C1	AYSKPTIKLANP	0.496±0.758	5-3-C1
3	5-3-E8	AYSKPTIKLANP	na	/
3	5-3-C5	AYSKPTIKLANP	na	/
3	5-3-B9	LPLATADKNGRT	0.436±0.624	5-3-B9
3	5-3-A4	DKLDNPGGPTVG	0.291±0.404	5-3-A4
3	5-3-E5	DKLDNPGGPTVG	na	/
3	5-3-A6	LQMPHNSKTANP	0.261±0.423	*
3	5-3-G6	INGQFSLKYRNP	0.253±0.188	5-3-G6
3	5-3-D4	LMPNKISNFASA	0.209±0.144	/
3	5-3-A1	DQTCNSPPCPPL	0.186±0.156	/
3	5-3-F7	STLPPPQGKIIH	0.185±0.243	/
3	5-3-B8	WYPLHSGLRSYY	0.156±0.121	/
3	5-3-B6	NKSTTNDFLRSP	0.141±0.122	/
3	5-3-D6	NGDYLQYKAPNP	0.139±0.141	/
3	5-3-B5	NTVRPPTLFYHW	0.126±0.109	/
3	5-3-D5	WHSEYQEPYPLS	0.120±0.103	/
3	5-3-A3	LDKNVLSPPMPL	0.107±0.082	/
3	5-3-C7	HHMSWYSRWLPV	0.091±0.071	/
3	5-3-A8	WWKPWSNFYGST	0.085±0.060	/
3	5-3-B3	LNTQNHAPLPSI	0.080±0.057	/

na: not applicable, SD: standard deviation,

*: not withheld: similar to 5-3-C1.

The phage clones were selected after 1, 2 or 3 positive selections. The peptide sequences that were expressed by these phage clones are given in column three. The average OD_c_ in sandwich ELISA, using nine purified antibody fractions as capture antibody, is shown in column four. If the peptide sequence was synthesised, the name of the biotinylated peptide is given in column five.

**Figure 2:**
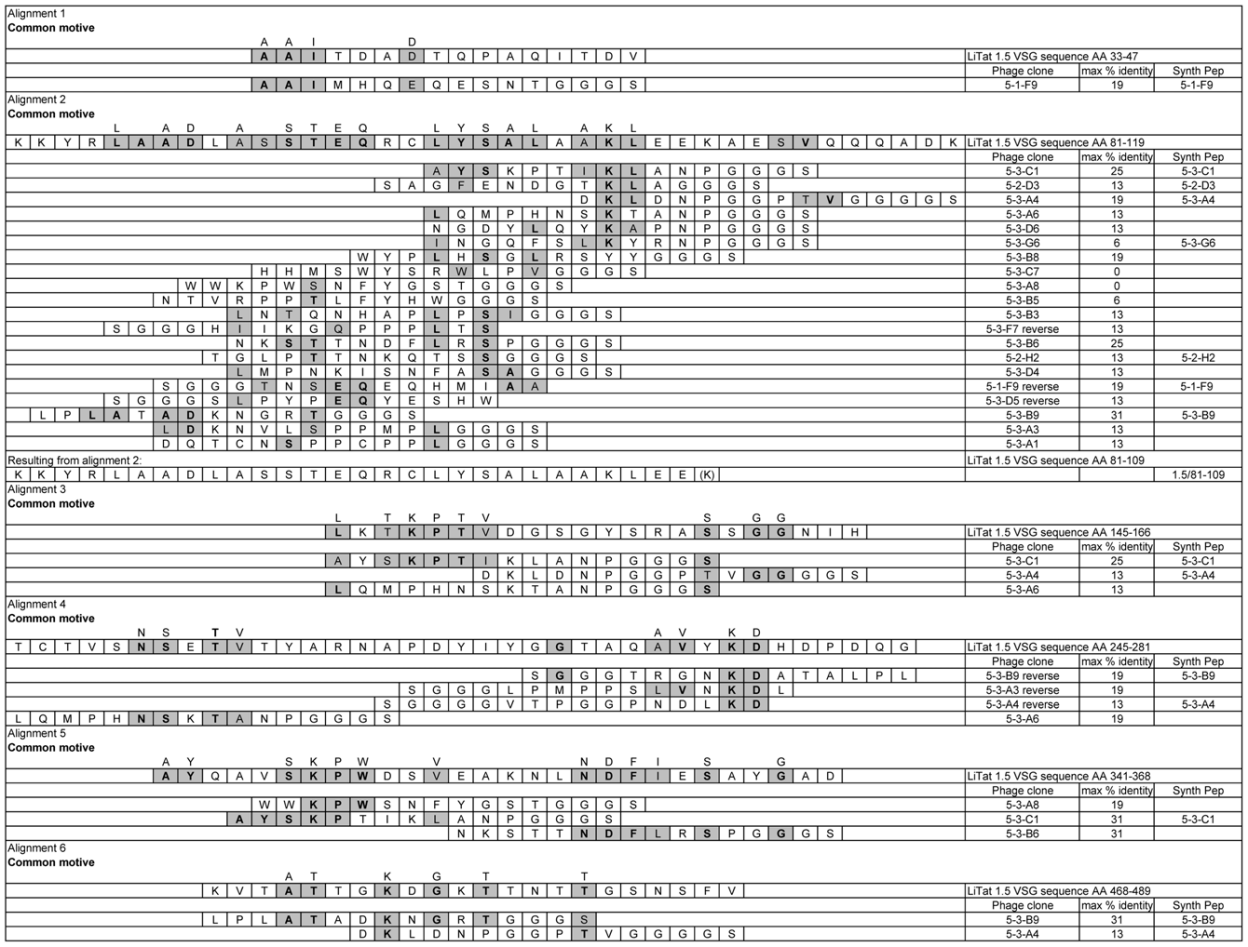
Alignment on VSG LiTat 1.5 of peptides selected with anti-VSG LiTat 1.5 antibody fractions. Homologous sequences between phage displayed peptides and/or the protein sequence of VSG LiTat 1.5 are indicated in grey. Amino acids that are identical to those of the VSG protein sequence are in bold and grey. All peptide sequences include the GGGS-spacer at the C-terminus. Maximum % identity: percentage identity of the peptide sequence with a corresponding stretch of sixteen AA within the protein sequence of VSG LiTat 1.5. Synth peptide: name of the synthesised peptide.

The alignment results of VSG LiTat 1.5 [GenBank HQ662603] and the 20 peptide sequences displayed by the phage clones are presented in figure 2.

The peptide expressed by phage clone 5-1-F9 (19% identity) could be aligned within AA 33 to 47 (alignment 1).

Within the N-terminal domain, 18 phage peptides could be aligned with minimum 6% identity within AA 81 to 119 (alignment 2), by analogy with alignment 1 for VSG LiTat 1.3. The peptides expressed by clones 5-3-C7, 5-3-A8 and 5-3-B5 respectively had 0, 0 and 6% identity within this AA stretch, but shared the common motive (W/F)Y with the peptide expressed by phage 5-3-B8 (19% identity). The peptide of phage clone 5-3-G6 had only 1/16 AA (6%) identity with VSG LiTat 1.5 but two more AA were homologous within this region. The reverse sequences of phage clone peptides 5-3-F7 (13% identity), 5-1-F9 (19% identity) and 5-3-D5 (13% identity) could also be aligned within this VSG LiTat 1.5 region. The peptide expressed by clone 5-3-B9 had the highest identity within the AA 81 to 119 stretch (5/16 AA, 31% identity, if a gap of 1 AA was allowed).

Peptides expressed by phage clones 5-3-C1, 5-3-A4 and 5-3-A6, with common motive “KLANP”, could also be aligned between AA 145 to 166 of the VSG LiTat 1.5 protein sequence (alignment 3) with respectively 25, 13 and 13% identity. Within the VSG LiTat 1.5 AA stretch 245 to 281, the peptide expressed by phage clone 5-3-A6, showed 19% identity and the reverse peptide sequence of phages 5-3-B9, 5-3-A3 and 5-3-A4 showed respectively 19, 19 and 13% identity (alignment 4). Within the boundary with the C-terminal domain of VSG LiTat 1.5, showed the peptides expressed by phage clones 5-3-A8, 5-3-C1 and 5-3-B6 respectively 19, 31 and 31% identity within AA stretch 341 to 368, if a gap of three AA was allowed for peptide 5-3-C1 (alignment 5). Within the C-terminal domain of VSG LiTat 1.5, phage clone peptide 5-3-B9 and 5-3-A4 had respectively 31 and 13% identity between AA 468 to 489 (alignment 6).

All selected phage clones were tested in a sandwich ELISA with the individual purified antibody fractions (table 2). The peptides displayed by the seven phage clones with the highest average OD_c_, were chosen for synthesis as biotinylated peptides, except for the peptide displayed by phage clone 5-3-A6, which was similar to 5-3-C1 but had a lower average OD_c_. Based on the alignment results and by analogy with VSG LiTat 1.3, the AA stretch 81 to 109 of the protein sequence of VSG LiTat 1.5 was synthesised as biotinylated peptide (peptide 1.5/81-109).

The reactivity of all eight biotinylated synthetic peptides was evaluated in indirect ELISA with the nine sera used for affinity antibody purification and with nine endemic negative controls. Peptide 5-3-A4 and 5-3-G6 had the lowest average OD_c_s (0.145 and 0.109) and shared a common motive with peptide 5-3-C1 with a higher average OD_c_ (0.289) and were therefore not withheld for testing on diagnostic performance. Peptide 1.5/81-109 had an OD_c_ of 0.382 and was withheld.

### Assessment of the performance of the biotinylated peptides for diagnosis of gambiense HAT

The accuracy of the biotinylated peptides to detect VSG-specific antibodies was assessed with sera from 102 *gambiense* HAT patients and 102 endemic negative controls (table 3). Among the mimotopes for VSG LiTat 1.3, the highest AUC was obtained with peptide 3-2-G5 (0.93) and peptide 3-2-G10 (0.95). Sensitivities and specificities at the cut-off with the highest Youden index were respectively 0.85 and 0.94 for peptide 3-2-G5, and 0.90 and 0.93 for peptide 3-2-G10. Of the mimotopes for VSG LiTat 1.5 the highest AUC was obtained with peptide 5-1-F9 (0.95) and 5-2-D3 (0.94) with respective sensitivities and specificities of 0.94 and 0.95 for peptide 5-1-F9 and 0.92 and 0.89 for peptide 5-2-D3. With peptide 1.3/78-110, an AUC of 0.95 was observed, with a sensitivity of 0.96 and a specificity of 0.85. With peptide 1.5/81-109, an AUC of 0.79, a sensitivity of 0.81 and a specificity of 0.75 were obtained.

**Table 3:** Evaluation of the potential of the biotinylated peptides for diagnosis of *T.b. gambiense* HAT.

Antigen type	Name	AUC (95% CI)	sensitivity (95% CI)	specificity (95% CI)
LiTat 1.3	1.3/78-110	0.95 (0.91–0.98)	0.96 (0.90–0.99)	0.85 (0.77–0.92)
LiTat 1.3	3-2-G10	0.95 (0.91–0.97)	0.90 (0.83–0.95)	0.93 (0.86–0.97)
LiTat 1.3	3-2-G5	0.93 (0.89–0.96)	0.85 (0.77–0.92)	0.94 (0.88–0.98)
LiTat 1.3	3-3-E3	0.89 (0.84–0.93)	0.96 (0.90–0.99)	0.76 (0.67–0.84)
LiTat 1.3	3-3-F6	0.89 (0.84–0.93)	0.82 (0.74–0.89)	0.86 (0.78–0.92)
LiTat 1.3	3-2-C5	0.89 (0.84–0.93)	0.90 (0.83–0.95)	0.82 (0.74–0.89)
LiTat 1.3	3-2-D10	0.86 (0.81–0.91)	0.86 (0.78–0.92)	0.81 (0.72–0.88)
LiTat 1.5	5-1-F9	0.95 (0.91–0.97)	0.94 (0.88–0.98)	0.95 (0.89–0.98)
LiTat 1.5	5-2-D3	0.94 (0.90–0.97)	0.92 (0.85–0.97)	0.89 (0.82–0.94)
LiTat 1.5	5-2-H2	0.88 (0.82–0.92)	0.82 (0.74–0.89)	0.81 (0.72–0.88)
LiTat 1.5	5-3-C1	0.87 (0.82–0.92)	0.86 (0.78–0.92)	0.79 (0.70–0.87)
LiTat 1.5	5-3-B9	0.85 (0.79–0.89)	0.79 (0.70–0.87)	0.83 (0.75–0.90)
LiTat 1.5	1.5/81-109	0.79 (0.73–0.85)	0.81 (0.72–0.88)	0.75 (0.65–0.83)
Native VSG	LiTat 1.3	1.000 (0.982–1.000)	1.000 (0.964–1.000)	1.000 (0.964–1.000)
Native VSG	LiTat 1.5	0.997 (0.973–1.000)	1.000 (0.964–1.000)	0.990 (0.947–1.000)

The ability of biotinylated synthetic peptides to bind human serum antibodies in 102 HAT positive and 102 endemic negative control sera was assessed by indirect ELISA. The area under the receiver operator characteristics curve (AUC) and the sensitivity and specificity at maximum Youden index are shown with 95% confidence intervals (CI).

VSG LiTat 1.3 and 1.5 obtained an area under the curve of respectively 1.000 and 0.997. The sensitivity and specificity were both 1.000 at cut-off 1.318 for VSG LiTat 1.3 and 1.000 and 0.990 at cut-off 1.182 for VSG LiTat 1.5.

### Three dimensional epitope mapping of the peptides with diagnostic potential

**Figure 3:**
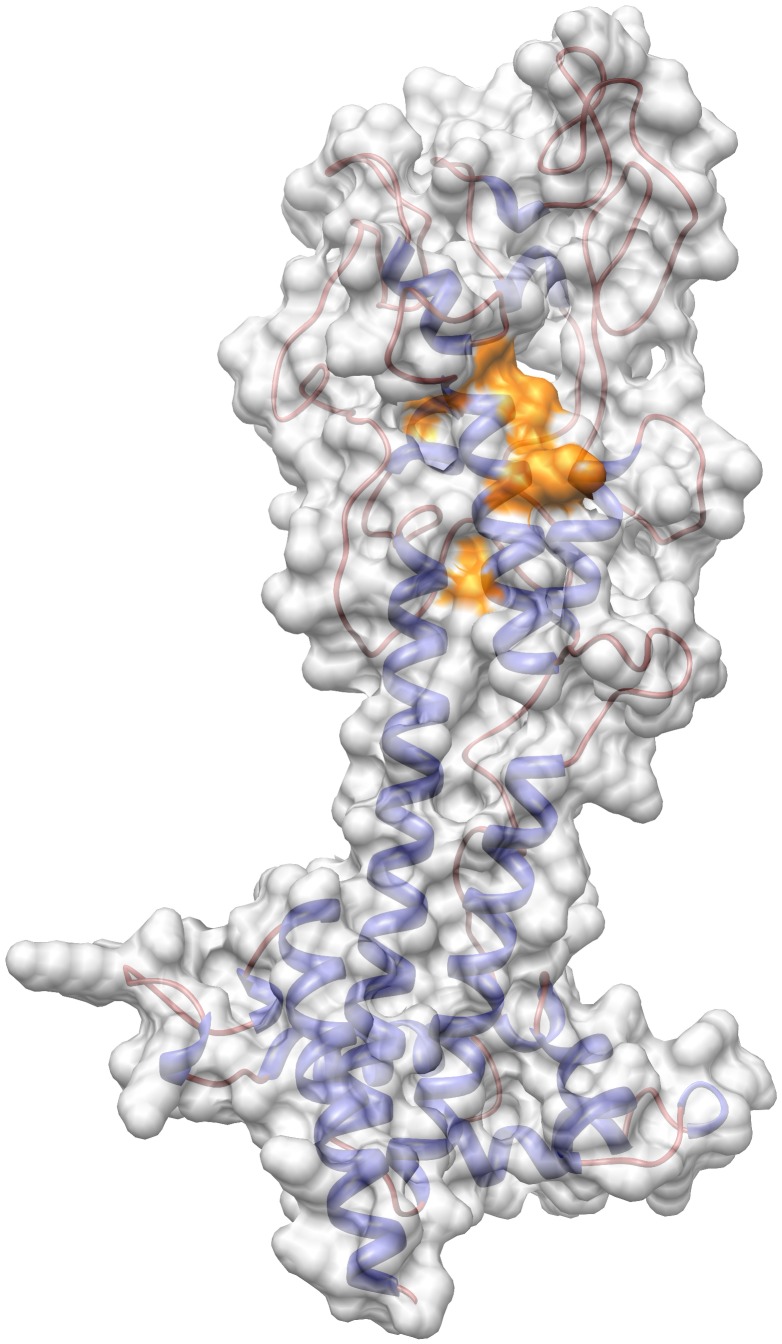
Mapping of peptide 5-2-D3. Peptide 5-2-D3 could be mapped (orange) with AA E 168|N 164|D 152|G 153|T 150|K 146|L 144|A 141 on the three-dimensional model of a VSG LiTat 1.5 N-terminal domain monomer by means of 3DEX and Chimera.

By using 3DEX software and setting the number of hits at a minimum of 5 AA, none of the VSG LiTat 1.3 mimotopes with AUC>0.90 could be mapped as a conformational epitope on the protein model of the VSG LiTat 1.3. In contrast, among the VSG LiTat 1.5 mimotopes with AUC>0.90, peptide 5-2-D3 could be mapped with 8/12 AA (E 168|N 164|D 152|G 153|T 150|K 146|L 144|A 141) on the three-dimensional VSG LiTat 1.5 protein model (figure 3).

## Discussion

In this manuscript we describe how mimotopes and regions that take part in epitope formation for VSGs LiTat 1.3 and LiTat 1.5 of *T.b. gambiense* were identified by screening of a Ph.D.-12 phage display library with polyclonal antibodies that were purified from sera of sleeping sickness patients.

As sera from sleeping sickness patients contain an important fraction of trypanosome unrelated antibodies [31], the risk of selecting mimotopes that are unrelated to sleeping sickness by using human sera for the screening was considerable.

We identified a linear region between amino acid 72 and 114 of the protein sequence of both VSG LiTat 1.3 and LiTat 1.5 wherein most of the peptide sequences could be aligned with the VSG protein sequence. This region is localised in the hypervariable N-terminal domain of the VSG and was for both VSGs synthesised as a linear biotinylated peptide and tested in indirect ELISA with a panel of 102 HAT positive and 102 endemic negative sera.

Peptide 1.3/78-110, corresponding to AA stretch 78 to 110 of VSG LiTat 1.3, had an AUC of 0.95, indicating diagnostic potential. The epitope of VSG LiTat 1.3, recognised by the human serum antibodies used for screening of the peptide library, therefore seems to be linear and located within AA stretch 78 to 110. The peptide sequences that were selected for VSG LiTat 1.3 had in average 3/16 amino acids in common within AA 72 to 114 with a maximum of 7/16 (44%) identical amino acids. Interestingly, a common motive of the peptide sequences was repeated twice within AA 72 to 114 of VSG LiTat 1.3: (F/W)ExDxK(A/L/V)x(A/L/V), from AA 77 to 85 and 101 to 109.

The two mimotopes of VSG LiTat 1.3 with the highest AUC, peptide 3-2-G5 and 3-2-G10, seemed to share a common epitope (correlation coefficient of OD_c_s with human sera in ELISA 0.71, data not shown), their motive WxxDxK reoccurred twice within AA 72 to 114. Their motive, (I/V/A)(T/S)DSK, could also be aligned within the C-terminal domain (AA 424 to 439). This AA stretch was synthesised as a biotinylated peptide as well, but had a low average OD_c_ upon a first screening with nine HAT sera and was discarded for further evaluation of diagnostic performance. We think it unlikely that peptide 3-2-G5 and 3-2-G10 are mimotopes for a linear epitope localised in the C-terminal domain of VSG LiTat 1.3. Additionally, epitopes localised in the relatively conserved C-terminal domain are more likely to react with non-VSG-specific antibodies.

As for VSG LiTat 1.3, a repetitive motive was present within AA 81 to 114 of VSG LiTat 1.5: (Y/F/W)(x or xx)(A/L/I/V)A(A/I/L)(D or K) (A/L)xxxxE, from AA 83 to 94 and AA 99 to 111. The peptide sequences selected for VSG LiTat 1.5 had in average 2/16 AA in common within AA 82 to 114 of the protein sequence, with a maximum of 5/16 (31%) identical AA. Contrary to the results for peptide 1.3/78-110, peptide 1.5/81-109, corresponding to AA stretch 81 to 109 of VSG LiTat 1.5, had an AUC of only 0.79, while the AUC of all of the individual peptides aligned in this region was >0.85. Motive AYSxxxIKL of peptide 5-3-C1 (AUC 0.87), corresponded to LYSxxxAKL (AA 99 to 106) of the VSG LiTat 1.5 protein sequence. Peptide 5-3-C1 seems therefore to mimic an epitope that is, albeit partly, localised in this region. The similar peptide 5-2-D3, with motive F(x)xxxxKL, performed better in ELISA (AUC 0.94). It is possible that peptide 5-2-D3 and 5-3-C1 are mimotopes for a discontinuous epitope as they share the common motive (A/I/L) (Y/F) xxxxxKLANPG with four other peptides, while ANPG was not found in the VSG protein sequence. We therefore suspect the epitope of VSG LiTat 1.5, recognised by the human serum antibodies used for screening, to be discontinuous and to be at least partly localised within this region. This might explain the weaker performance of the linear peptide 1.5/81-109 compared to the mimotope peptides. This finding was supported by the results of the 3DEX analysis of the mimotopes that had an AUC>90, locating peptide 5-2-D3 with 8/12 AA on the three-dimensional VSG LiTat 1.5 protein model (E 168|N 164|D 152|G 153|T 150|K 146|L 144|A 141).

In a previous study [16] we were able to identify mimotope peptides for the native trypanosomal variant surface glycoproteins by screening of peptide phage display libraries with monoclonal antibodies. Through phage display with polyclonal human antibodies we now identified different mimotopes and regions taking part in epitope formation. Because the three monoclonal antibodies used in the first study represent only a fraction of the VSG-specific antibody response, some mimotopes with diagnostic potential might have been missed. Additionally, the mouse and human immune system may recognise different B cell epitopes. Other factors may have contributed to finding different motives using the two approaches. As a result of a short infection period of two weeks, the mouse monoclonals do not recognise all VSG-epitopes. They were selected for strict VAT-specificity with purified VSGs and identified mimotopes, not necessarily dominant, that were located near the surface of the VSG N-terminal domain. The polyclonal human antibodies result from a long infection and recognise also less exposed VSG-epitopes. It may be that by affinity purification on purified VSG an antibody fraction that recognises non-surface epitopes was mainly retained, as the mimotopes of VSG LiTat 1.3 seem to be located in this region. Another explanation may lie in the presence of self-reactive VSG-specific antibodies in sera from uninfected individuals, as has been demonstrated by Müller et al. [32]. Thus the negative selection with human antibodies from control sera may have eliminated the phages expressing the mimotopes for the VSG-specific epitopes also recognised by the mAbs. Both panning strategies thus seem complementary, in contrast to what has been described by Tang *et al.* [18], who selected a greater number of different 12-mer sequences with polyclonal serum for *Salmonella enterica*, but some of the common motives were also selected by panning of a monoclonal antibody. In the manuscript of Casey *et al.* [17] the mimotopes for Epstein-Barr (EBV) virus, selected with polyclonal EBV immune rabbit and patient sera were also not recognised by the monoclonal antibodies used for mimotope selection in a previous study.

Diagnostic evaluation of individual mimotopes and combinations [patent application GB1202460.0] indicates that screening of peptide phage display libraries with patient's antibodies resulted in a more efficient selection of diagnostic peptides than with monoclonal antibodies. We therefore prefer screening with patient's antibodies. As an alternative approach to phage display linear epitopes may be replaced by synthetic peptides identified by scanning of overlapping peptides spanning the native protein sequence [33]. Furthermore, there are alternative *in vitro* methods for phage display such as yeast cell or bacterial display or, non-cellular, ribosome or mRNA display [34].

Our study has nevertheless some limitations. First, considering the broad antibody spectrum in HAT sera due to polyclonal B cell activation [35], we opted to use antibody fractions that were affinity purified for VSG LiTat 1.3 and 1.5. Thus, mimotopes for other predominant VSGs or other trypanosome antigens with diagnostic potential have not been selected. An alternative approach to identify additional diagnostic mimotopes may consist of screening peptide phage libraries with patient antibodies against other candidate diagnostic proteins [36]. Examples are the *T.b. gambiense*-specific glycoprotein TgsGP [37], the *T.b. rhodesiense*-specific serum resistance associated (SRA) protein [38] and *Trypanozoon*-specific trypanosome antigens such as invariant surface glycoprotein (ISG) 65 and ISG 75 [39], microtubule associated repetitive protein 1 (MARP1) and GM6 [40]. Some of them have already been tested for their diagnostic potential in the form of recombinant fusion proteins expressed in *E. coli* but none are yet used in diagnostic tests for HAT. Second, even with affinity purified antibodies there is a risk that non-specific mimotopes are selected with antibodies against VSG epitopes that are normally hidden in the intact VSG coat. Usually, most of the phage particles display a consensus binding sequence after two or three rounds of enrichment [21]. We therefore performed three rounds of positive selection and two selections with negative sera. Remarkably, the mimotopes with the highest AUC for VSG LiTat 1.3 and 1.5 were selected after only two or even one round of panning. Third, no affinity measurements *e.g.* via surface plasmon resonance, have been performed. Considering the polyclonal character of antibodies in patients' sera and the inherent differences in antibody response between individual patients, we opted to assess only the diagnostic potential of the selected peptides by means of ELISA.

Before the native *T.b. gambiense* VSGs LiTat 1.3 and LiTat 1.5 in the currently existing diagnostic formats can be replaced by synthetic peptides, further improvements should be considered. It is possible to define critical residues, essential for binding with the antibody, by e.g. alanine scanning mutagenesis [41]. Thus the epitope of the human serum antibodies might be recreated as has recently been done for a linear epitope on the VP1 protein of foot-and mouth disease virus [42]. Other, non-essential, parts of the peptides can then be eliminated in order to increase specificity. Phage clones that express peptides with a higher binding affinity might be selected by increasing the number of selection rounds and/or the stringency of the washing steps.

In conclusion, with this study we demonstrate that mimotopes of *T.b. gambiense* VSG LiTat 1.3 and 1.5 can be selected from a phage display library and that these mimotopes and corresponding amino acid stretches within the VSGs have diagnostic potential.
